# Scoping review of *Culex* mosquito life history trait heterogeneity in response to temperature

**DOI:** 10.1186/s13071-023-05792-3

**Published:** 2023-06-14

**Authors:** S. Kane Moser, Martha Barnard, Rachel M. Frantz, Julie A. Spencer, Katie A. Rodarte, Isabel K. Crooker, Andrew W. Bartlow, Ethan Romero-Severson, Carrie A. Manore

**Affiliations:** 1 Genomics and Bioanalytics (B-GEN), Los Alamos National Laboratory, Los Alamos, NM USA; 2 Information Systems and Modeling (A-1), Los Alamos National Laboratory, Los Alamos, NM USA; 3 Department of Biostatistics, School of Public Health, University of Minnesota Twin Cities, Minneapolis, MN USA; 4 Department of Biology, Cornell University, Ithaca, NY USA; 5 Theoretical Biology and Biophysics (T-6), Los Alamos National Laboratory, Los Alamos, NM USA; 6 Department of Mathematics and Statistics, Utah State University, Logan, UT USA

**Keywords:** West Nile virus, Culex, Culicidae, Arboviruses, Temperature, Climate change

## Abstract

**Background:**

Mosquitoes in the genus *Culex* are primary vectors in the US for West Nile virus (WNV) and other arboviruses. Climatic drivers such as temperature have differential effects on species-specific changes in mosquito range, distribution, and abundance, posing challenges for population modeling, disease forecasting, and subsequent public health decisions. Understanding these differences in underlying biological dynamics is crucial in the face of climate change.

**Methods:**

We collected empirical data on thermal response for immature development rate, egg viability, oviposition, survival to adulthood, and adult lifespan for *Culex pipiens, Cx. quinquefasciatus, Cx. tarsalis*, and *Cx. restuans* from existing literature according to the PRISMA scoping review guidelines.

**Results:**

We observed linear relationships with temperature for development rate and lifespan, and nonlinear relationships for survival and egg viability, with underlying variation between species. Optimal ranges and critical minima and maxima also appeared varied. To illustrate how model output can change with experimental input data from individual *Culex* species, we applied a modified equation for temperature-dependent mosquito type reproduction number for endemic spread of WNV among mosquitoes and observed different effects.

**Conclusions:**

Current models often input theoretical parameters estimated from a single vector species; we show the need to implement the real-world heterogeneity in thermal response between species and present a useful data resource for researchers working toward that goal.

**Graphical Abstract:**

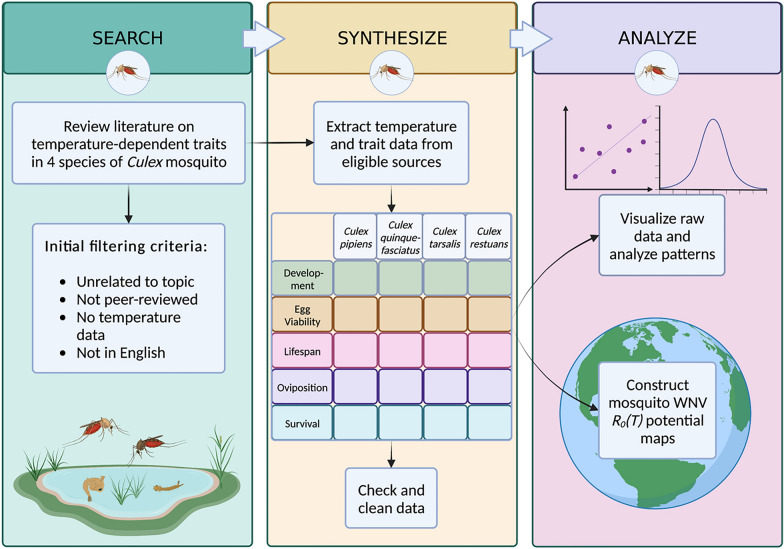

**Supplementary Information:**

The online version contains supplementary material available at 10.1186/s13071-023-05792-3.

## Background

The temporal and spatial extents of arthropod disease vector distributions are largely determined by climate and other environmental conditions [1, 2]. As climate conditions change, vectors respond by expanding or shifting their ranges and adapting to new environments [3]. Current efforts to predict the range of vectors and the risk of vector-borne diseases in the future require many quantified variables, including knowledge of how these vectors will respond to changes in temperature and precipitation patterns [4–6] and photoperiod for diapausing species [7]. Vector range expansion can occur simply because vectors are tracking suitable conditions through dispersal and colonization [3, 8]. Temperature alone plays a role in whether vectors like mosquitoes can overwinter in a given area, which contributes to their persistence in new regions [9]. Mosquitoes are ectothermic, meaning their ability to regulate their own temperature is very limited; therefore, they are sensitive to the effects of temperature at all stages of their life cycle [10]. Temperature variation also influences disease transmission [11].

Mosquitoes in the genus *Culex* are the primary vectors for a range of dangerous diseases, notably West Nile virus (WNV), St. Louis encephalitis virus, Japanese encephalitis virus, western equine encephalitis virus, Sindbis virus, Rift Valley fever virus, and filarial parasites causing human lymphatic filariasis [12–18]. *Culex* mosquitoes have recently undergone range expansions in North America [19] and will likely continue to do so in the future [20]. As mosquito species expand and shift their geographic ranges, the pathogens they harbor expand and shift as well [21], resulting in new challenges for previously unaffected regions. For example, *Culex quinquefasciatus* is mainly found in the tropics and subtropics, while *Culex pipiens* is found in more temperate regions. Both species are predicted to increase their range northward into Canada and to subsequently increase the risk of WNV and other arboviruses [20, 22]. Species distribution models are key for understanding the current ranges of *Culex* species important for human disease spread [23] and for predicting future spread under varied climate scenarios [24].

Mosquito responses to environmental conditions are variable and frequently stray from model predictions of how changing climate will determine future distributions [6]. There are three primary reasons for this: (i) lack of systematic data for model parameterization; (ii) innate biological heterogeneity across mosquito species; (iii) difficulty in capturing that heterogeneity within the framework of even the most complex model. As with any undertaking in ecological science, it is difficult to produce observations of natural mosquito behaviors that can be confidently assigned to causative relationships, because the interdependencies between organism and environment are often nonlinear. For example, Cx. *quinquefasciatus* mosquitoes are highly adapted to urban environments, giving them the ability to survive beyond their distribution ranges [25, 26]. Additionally, confusion exists in the taxonomic classification of *Culex* mosquito species themselves. For example, *Cx. quinquefasciatus* was widely considered a subspecies of the *Cx. pipiens* complex until 1978 and referred to as *Culex pipiens fatigans* and *Cx. p. quinquefasciatus* interchangeably [27]. Since then, it has been regarded as distinct along with several other former *Cx. pipiens* subspecies; however, reporting in the literature is often inconsistent given that the exact composition of the *Cx. pipiens* complex and its phylogeny remains ambiguous, and hybridization has been reported [27, 28].

In terms of the variability in mosquito population dynamics and how this affects broader systems related to ecosystem function and public health, it is important to remember that the effects of climate change are unpredictable. Beyond global increases in average temperatures, extreme weather events may cause mosquito populations to behave erratically with unforeseen consequences. For example, the adult lifespan of *Cx. pipiens* is negatively correlated with temperature within the bounds of upper and lower survival thresholds. *Culex pipiens* abundance is predicted to decrease with hotter summers [29], and WNV transmission could potentially be reduced as a result [30]. In contrast, rising minimum winter temperatures have been shown to prolong host-seeking and oviposition behavior in *Cx. pipiens*, likely leading to a longer WNV transmission season and overall more resilient mosquito populations [7]. The variety of mosquito overwintering mechanisms also contributes to this resiliency, making them better equipped to withstand some of the less obvious consequences of climate change such as sudden bouts of unseasonably cold weather and decreased winter rainfall [7]. WNV exists in nature through a continuous cycle of mosquito to bird to mosquito transmission and spills over to cause disease in humans via infected mosquito bites [14, 31]. Since its first isolation in Uganda in 1937, it spread and quickly became established on new continents and has become endemic to North America [32]. This is largely due to the adaptability of *Culex* mosquitoes, their efficiency as vectors, and an abundance of competent avian hosts to amplify the virus [12, 31, 33]. Accurate model predictions of WNV dynamics are not only valuable for WNV mitigation, but also for understanding and mitigating new and emerging zoonotic diseases with similar modes of transmission [34–36].

The task of understanding the interactions between mosquito life history traits and environmental variables and applying them to quantify disease risk with any degree of accuracy is made difficult by trait-, species-, and even population-specific effects [37]. Despite this, the subject has grown as a research priority, from early work describing sporozoite rate (the percentage of mosquitoes with *Plasmodium* sporozoites present in their salivary glands) to identification of specific mosquito life history traits that have the highest impact on disease transmission [38]. This has informed data-driven calculations of the basic reproductive number, *R*_0_, defined as the expected number of secondary infections in a susceptible population resulting from a single infectious individual, also referred to as vectorial capacity [39–41]. Various methods of estimating this quantity have been established that incorporate mosquito life history traits and their dependence on environmental variables [11, 41–44]. A related concept to *R*_0_ is the type reproduction number, here denoted $R_0^T$, which is defined as the expected number of secondary infections in a susceptible population of one type caused by a single infectious individual of the same type [45]. It was developed to improve the performance of *R*_0_ in heterogeneous systems where more than one type of host is important to the transmission cycle, and the differences between them are epidemiologically significant [45]. In the case of WNV as explored here, this translates to a threshold quantity for the expected transmission of WNV from mosquito to mosquito. As a relatively simple metric of infection potential among distinct vectors that can be calculated without employing a complex transmission model, $R_0^T$ is a an interesting way to illustrate important biological differences between *Culex* mosquito species in relation to key climate drivers such as temperature.

To inform calculations of transmission potential in enzootic cycles, and thereby support efforts to understand risk to humans, empirical data are needed that quantify the underlying biological heterogeneity of mosquito species. To address this need, we conducted a scoping review of the literature for experimentally derived data from peer-reviewed studies on the empirical relationships between temperature and five life history traits in four *Culex* mosquito species. We examined trends in the literature-derived data and applied a simple calculation of WNV mosquito $R_0^T$ adapted from an equation previously reported to model temperature-dependent WNV *R*_0_ [46] to estimate the type reproduction number for temperature-dependent endemic spread of WNV among mosquitoes, $R_0^T$, and demonstrate the impact of vector species differences on estimates of WNV transmission potential in North America. In view of changing temperatures, shifting vector ranges, and the expanding endemicity of WNV, the data, trends, and conclusions presented here are useful resources for researchers working to understand and implement how underlying heterogeneity in vector biology affects population dynamics and disease risk factors in the face of climate change.

## Methods

### *Culex* mosquito species and life history traits

We focused our analysis on four *Culex* species found across North America: *Culex pipiens, Cx. quinquefasciatus, Cx. tarsalis*, and *Cx. restuans*. We chose these species because of their epidemiological relevance as disease vectors dominating different geographical areas and ecological niches [43, 47, 48]. *Culex pipiens* and *Cx. restuans* are considered to be the primary amplification vectors for WNV within their distribution areas [12, 49, 50]. *Culex pipiens* is distributed in the midwestern, northern, and eastern US, with high habitat suitability in the northeast, Great Lakes area, parts of California, and the northwest [23], as well as in parts of Mexico [51] and Canada [20]. *Culex restuans* is distributed across the eastern half of the US, with high habitat suitability along the entire east coast as well as the Great Lakes area [23]. Highly competent *Culex tarsalis* and *Cx. quinquefasciatus* are likely responsible for elevated risk of WNV infection in humans west of the Mississippi River, with *Cx. quinquefasciatus* often driving transmission in urban areas and *Cx. tarsalis* driving transmission in rural areas as well as suburban areas near irrigated agriculture or wetlands [50]. *Culex quinquefasciatus* has high habitat suitability in the southwestern and southeastern US, Mexico, and South and Central America [23]. *Culex tarsalis* is distributed throughout the US, with extremely high habitat suitability in most of the country except the far south and southeast [23]. Although there are other species of mosquitoes in the genus *Culex* that act as vectors and have the potential to increase disease spread under climate change [23], we limited our study to the aforementioned four species.

**Fig. 1:**
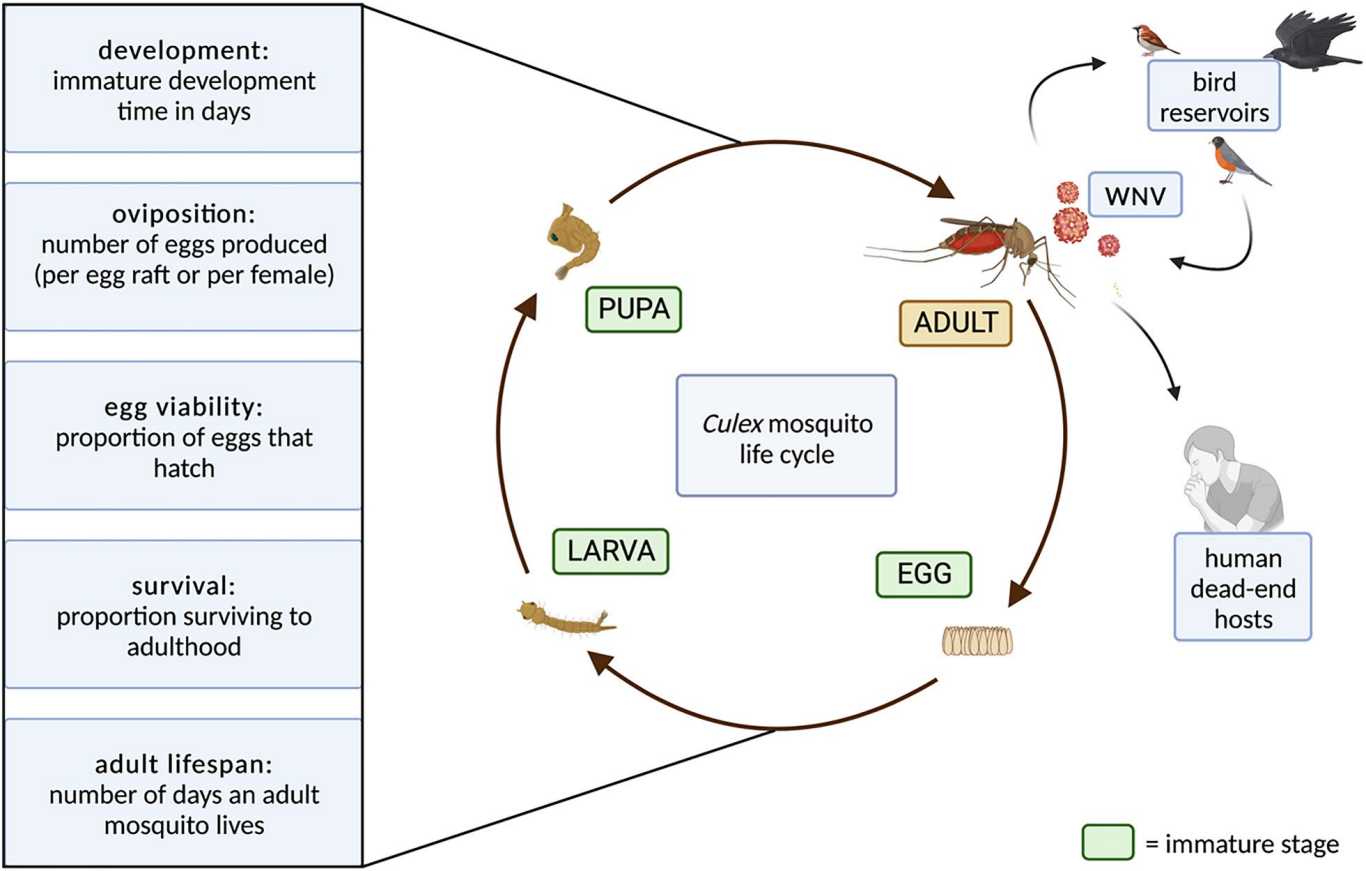
*Culex* mosquito life cycle, key traits, and their role in the West Nile virus (WNV) transmission cycle. Definitions of life history traits examined in this study (left), life stages (center), and enzootic circulation among mosquitoes and birds (top right)

For each species, we investigated five directly measurable life history traits with empirical values in the peer-reviewed literature at various temperatures. The traits examined were immature development (time in days to reach next life stage), survival to adulthood (percentage of eggs becoming adults), oviposition (number of eggs, either per egg raft or per female), egg viability (percentage of eggs that hatch), and adult lifespan (time in days an adult mosquito lives) (Fig. 1).

### Literature search

**Fig. 2:**
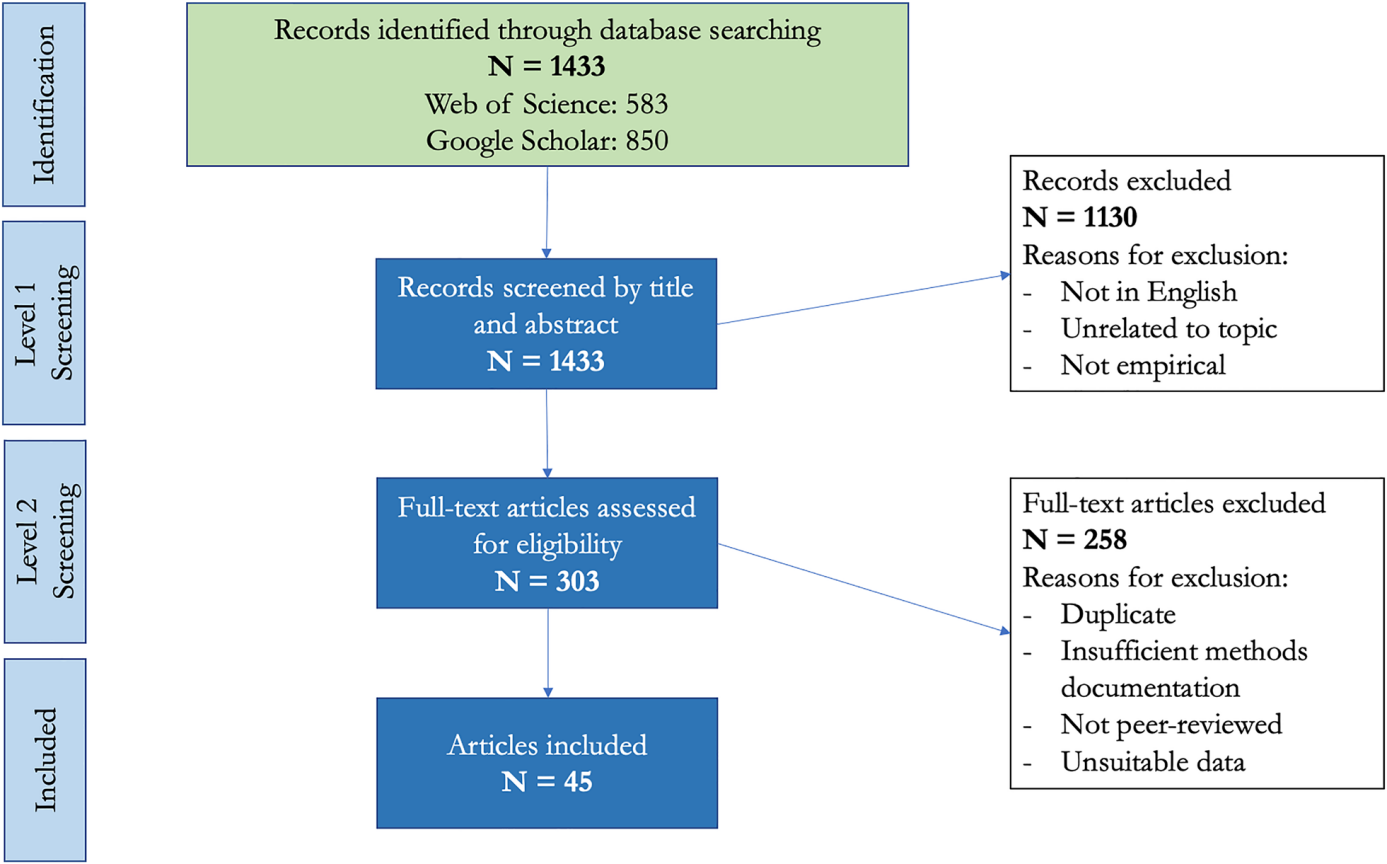
Literature search and filtering protocol. Identification level consisted of searching databases. Level 1 screening eliminated records by title and abstract according to Level 1 criteria, and Level 2 screening eliminated full-text articles after careful review according to Level 2 criteria

We used Web of Science as our primary search engine and Google scholar as a secondary source to confirm bibliographic data and locate additional sources. Our search included papers published from January 1950 to May 2022. Following the PRISMA Scoping Review guidelines [52], we kept a list of search terms for each database and recorded the number of initial hits and number of remaining records after each filtering step for each species and trait (Additional file 3: Dataset S3). In Web of Science, our primary database, we searched for titles and abstracts containing the species name, the name of the parameter, and temperature: for example, “TI = [(“*Culex quinquefasciatus*” OR “cx. quinquefasciatus”) AND (lifespan) AND (temperature)] OR AB = [(“*Culex quinquefasciatus*” OR “cx. quinquefasciatus”) AND (lifespan) AND (temperature)]. We read the titles and abstracts of all records found in Web of Science under these search terms and filtered out records based on the following criteria: not in English, unrelated to the topic, not peer-reviewed, or no mention of temperature (Fig. 2). We read the remaining full-text records carefully and eliminated those without empirical data relating temperature to the parameter (e.g. only summary statistics) and those without sufficient explanation of methods (e.g. no clear parameter definition or no specification of whether the study conditions were reasonably controlled) (Fig. 2).

We used Google Scholar as a secondary database to find additional records. The protocol was the same as for Web of Science, except that only the first 50 records in each search result were examined because the results consistently had little to no relevance past the first 20–30 (Additional file 3: Dataset S3).

Of 1433 initial hits through both Web of Science and Google Scholar, 1130 were removed in the primary filtering steps. Of 303 remaining records, 258 were filtered because of lack of suitable data, insufficient explanation of methods, lack of peer review, and removal of duplicates. Forty-five full-text studies were included in the review for data extraction.

### Data extraction and synthesis

From each of the 45 studies that met our criteria for data extraction, we systematically recorded the values and units for each life history trait, temperature at which the values were determined, standard error or standard deviation (if provided), range, geographic location of the study, and any relevant notes or significant findings. If the full dataset from a study was not publicly available or not provided, but values could be reasonably estimated from figures, this was done using the browser tool WebPlotDigitizer (Additional file 2: Dataset S2). For certain traits and species, the only temperature-associated data available were control values from studies primarily concerned with the effects of other variables. In these cases, a note was made, and the values were only included if the study presented truly controlled conditions (Additional file 2: Dataset S2). We reported results that included both air temperature and water temperature (Additional file 4: Dataset S4). The data were combined and cleaned, preserving the attached metadata (Additional file 1: Dataset S1). Plots were produced using R library ggplot2 [53, 54].

### West Nile virus mosquito $R_0^T$ potential

The basic reproduction number, $R_0$, represents the number of secondary infections created by a primary infection within a fully susceptible population [55]. This value both estimates transmissibility of a disease within a given population and captures the boundary of ann epidemic at $R_0 \geqslant 1$ [56]. One traditional parameter-driven formulation of *R*_0_ for malaria is $R_0 = \frac{{a^2 bce^{\frac{\mu }{\nu }} M}}{r\mu }$ ([40]; parameter descriptions are given in Table 1). However, this formulation does not include life history traits of mosquitoes and focuses on transmission parameters. In addition, while $M$, the mosquito to bird host density ratio, has a large impact on $R_0 ,$ estimates of this value are often not as informed by mosquito population data as they could be [57].

**Table 1:** R0 equation parameters

Parameter	Description	Value	Units	Source
$a$	Biting rate (mosquito to host)		Bites/day	[11]
$b$	Transmission probability mosquito to host		N/A	[11]
$c$	Transmission probability host to mosquito		N/A	[11]
$\mu$	Mortality rate mosquito		1/day	Data from literature (Additional file 1: Dataset S1)
$\nu$	Inverse of mosquito extrinsic incubation period		1/day	[11]
$r$	Inverse of host infectious period	0.20	1/day	[61, 62]
$M$	Mosquito to host density ratio		N/A	N/A
$B$	Birds per female mosquito	2	birds	Given based on assumptions
$D$	Natural bird death rate	0.0014	1/day	[62, 63]
$EF$	Eggs per female mosquito	131.77	eggs/female	Data from literature (Additional file 1: Dataset S1)
$EV$	Egg viability		N/A	Data from literature (Additional file 1: Dataset S1)
$pLA$	Proportion of larvae surviving to adulthood		N/A	Data from literature (Additional file 1: Dataset S1)

Description of each parameter, the value, and units for Eq. 1

We propose a modification of the above formulation for *R*_0_ where *M* is replaced by $EF\left( T \right)EV\left( T \right)pLA\left( T \right) \times \frac{1}{B} \times \frac{D}{\mu \left( T \right)}$ where the first term represents the temperature-varying number of eggs that survive to adulthood produced per female mosquito, the second term normalizes to the bird population, and the third term is the ratio of death rates of birds over mosquitoes. This allows us to calculate a quantity closely related to *R*_0_ known as the type reproduction number, $R_0^T$, designed to target heterogeneous systems such as WNV by estimating the number of secondary infections in a particular type of host or vector caused by an individual of that same type [45]. This quantity can be understood as the product of bird to mosquito transmission probability and mosquito to bird transmission probability, resulting in a metric for vector-vector transmission.

Here, we define “mosquito $R_0^T$ potential,” or $R_0^T$, as the potential number of secondary mosquito infections in a fully susceptible population caused by a single mosquito index case at a given temperature. Therefore, as a modification of the temperature-dependent equation for *R*_0_ previously reported in [11], replacing *M* and indicating which variables are temperature dependent, we have:

1 $$R_0^T = \frac{{a\left( T \right)^2 b\left( T \right)c\left( T \right)e^{\frac{\mu \left( T \right)}{{\nu \left( T \right)}}} EF\left( T \right)EV\left( T \right)pLA\left( T \right)D}}{rB\mu \left( T \right)^2 }.$$

All temperature-varying parameters as shown in Eq. 1 have been shown to change with temperature in previous experimental and modeling studies [11]. All parameter descriptions and values (for constant parameters) are in Table 1. Note that for simplicity, we assume the bird population is constant over time, such that the number of birds per female mosquito (*B*) is two. Additionally note that (i) for many pathogens the extrinsic incubation period (EIP) is also affected by temperature, with hotter conditions resulting in shorter EIPs [58], and (ii) though vertical transmission of WNV occurs [59, 60], we do not consider it here. Our formulation represents the enzootic vector-vector threshold only and does not factor in humans.

#### Statistical methods

To calculate mosquito $R_0^T { }$ for any temperature, we fit functions of temperature to each temperature-varying parameter. We fit a Brière [64], quadratic [65], or linear function for each model. Brière and quadratic functions were fit using the R package nls.multstart [66], while linear models were fit with the R package stats [53]. The Brière function models an asymmetrical unimodal thermal response, while the quadratic function models a symmetric unimodal response. We reviewed here only directly measurable temperature-varying mosquito life traits. For temperature-varying parameters not explored in the literature review but included in our analysis, we used data and methods from [11] to fit the temperature-varying functions. We fit the same functions that [11] used for each parameter [Brière function for biting rate ($a$) and inverse of the extrinsic incubation period ($\nu$); quadratic function for the probabilities of transmission ($b, c$)] on all *Culex* data [11] provided, not for each species, because of limited data for most species. We chose to use the same functions as [11] because they already validated these functions for the data. For parameters explored within the literature review, we fit functions by *Culex* species; linear, quadratic, or Brière was selected by Akaike information criterion (AICc). We performed this selection process because these functions have not been previously fit to the breadth of data collected in our literature review process. For lifespan $\left( {\frac{1}{\mu }} \right)$ and development rate, a linear function was the best fit, as also found in previous literature [11, 37, 67]. For egg viability and survival percentage, a quadratic function performed the best by AICc. We did not create a functional fit on the oviposition data because the data were too noisy to recover a reliable signal given the number of measurements.

We calculated mosquito $R_0^T$ *Cx. pipiens, Cx. quinquefasciatus*, and *Cx. tarsalis*, but not for *Cx. restuans* because of a lack of egg viability data. We obtained historical monthly average temperature data for the US from 2010 to 2020 from the ERA5-Land reanalysis dataset through the Copernicus Climate Data Store [68]. We averaged these data over the WNV season from May–September for all years and visualized it at the county level. We calculated mosquito $R_0^T { }$ potential for each individual species with input temperature data for two scenarios. First, current conditions calculated from the decadal county-level mean temperatures; second, hypothetical future conditions under an increase of 3 °C. Other inputs were the functional fits of the temperature-varying parameters (Additional file 5: Table S5) and the constant parameters in Table 1. All data collection was performed in Python 3.9 (Python Software Foundation, https://www.python.org), and all analyses and visualization were performed in R 4.2.33 [53].

## Results

### Literature review

Of 1433 initial search records that were filtered for relevance, empirical approach, and English language, 303 full-text studies remained. Of these 303, 45 met our inclusion criteria for being unique peer-reviewed studies with clear method documentation and suitable data: 1313 for *Cx. pipiens*, 18 for *Cx. quinquefasciatus*, 4 for *Cx. restuans*, and 12 for *Cx. tarsalis*.

The 13 *Cx. pipiens* publications that met our inclusion criteria included 4 field studies, 5 laboratory studies, and 4 that were unclear on setting. Of these 13 publications, 6 measured air temperature, 3 measured water temperature, and 4 were unclear. Temperatures recorded in *Cx. pipiens* studies ranged from 3 to 38 °C.

The 18 *Cx. quinquefasciatus* publications included consisted of 4 field studies, 10 laboratory studies, and 4 that were unclear on setting. Of these 18 publications, 7 measured air temperature, 5 measured water temperature, and 6 were unclear. Temperatures recorded in *Cx. quinquefasciatus* studies ranged from 15 to 40 °C. The four *Cx. restuans* publications included two field studies, no laboratory studies, and two that were unclear on setting. Of these four publications, two measured air temperature, none measured water temperature, and two were unclear. Temperatures recorded in *Cx. restuans* studies ranged from 15 to 33 °C.

The 12 *Cx. tarsalis* publications that met our inclusion criteria included 1 field study, 8 laboratory studies, and 3 that were unclear on setting. Of these 12 publications, 1 measured air temperature, 1 measured water temperature, and 10 were unclear. Temperatures recorded in *Cx. tarsalis* studies ranged from 13 to 37 °C.

#### *Culex pipiens*

*Culex pipiens* is considered to be a major vector of WNV in the northeastern and north central US [69] and the primary bridge vector that carries WNV from birds to humans [30, 70].

**Fig. 3:**
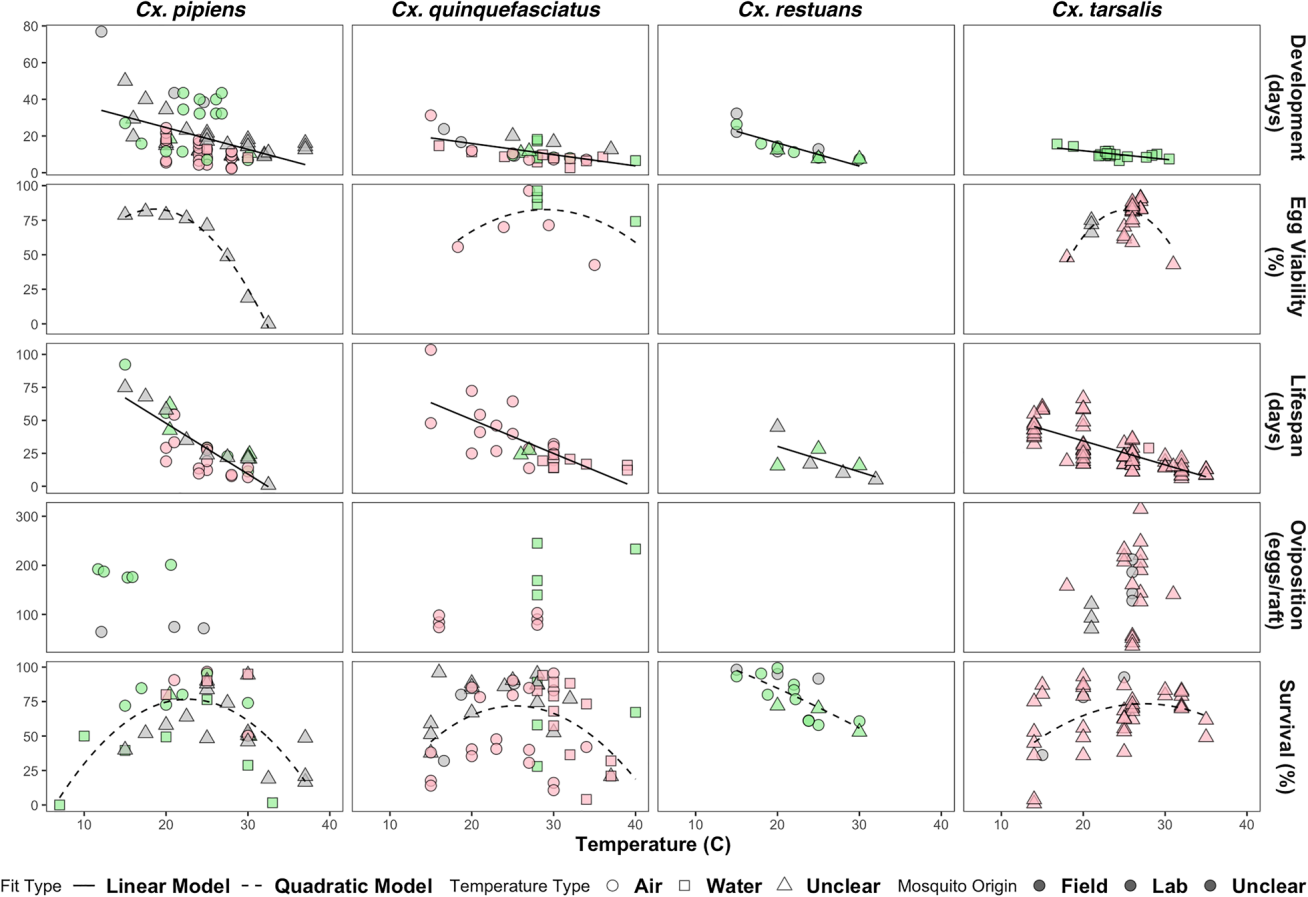
Life history trait response to temperature in four *Culex* mosquito species. Left to right: *Culex pipiens, Cx. quinquefasciatus, Cx. restuans*, and *Cx. tarsalis*; traits top to bottom: immature development time in days, percent egg viability, adult lifespan in days, oviposition in number of eggs per egg raft, and percent immature survival. Mosquito origin indicated by color: green = field collected, pink = laboratory colony, gray = unspecified or unclear. Temperature measurement type indicated by shape: circles = air temperature, squares = water temperature, triangles = unspecified or unclear. Type of fit indicated by line type: solid = linear model, dashed = quadratic model. See Table 2 for key summary values, Additional file 1: Dataset S1 for the full dataset used to generate this figure and Additional file 5: Table S5 for descriptions of the fitted functions

All papers reviewed reported that as temperature increased, *Cx. pipiens* development time decreased up to the maximum temperature studied (32.5 °C). Shortest development times were 2.3–10 days at temperatures from 30 to 32.5 °C, and longest development times were 25–50 days from 15 to 20 °C (Fig. 3, Table 2).

**Table 2:** Summary of temperature-dependent trait values from literature

Species	Trait	Min–Max	Mean ± SE	Median	References
*Culex pipiens*	Development	2.278–76.923	19.1 ± 1.607	15.629	[37, 71, 73, 75–82]
	Egg viability	0–81.2	56.7 ± 11.081	73.6	[71]
	Lifespan	1–92.3	30.374 ± 3.911	23.4	[30, 70–73, 75, 78]
	Oviposition^a^	64.5–201	142.826 ± 21.455	175.65	[71, 74–79]
	Survival	0–96.6	62.897 ± 4.909	72.35	[71, 72, 75, 77–80, 82, 83]
*Cx. quinquefasciatus*	Development	2.703–31.25	11.403 ± 1.04	9.392	[59, 67, 83–91]
	Egg viability	42.6–96.33	76.041 ± 6.201	74.14	[85, 87, 92, 93]
	Lifespan	12.2–103.5	33.018 ± 4.196	25.8	[59, 72, 90, 91, 94, 95]
	Oviposition^a^	74–245	131.508 ± 20.194	100.5	[85, 87, 90, 95–98]
	Survival	3.99–96	60.685 ± 3.42	67.115	[37, 67, 72, 85, 86–89, 91, 94, 95, 98, 99]
*Cx. restuans*	Development	7.299–32.258	14.102 ± 1.907	12.5	[79, 100, 101]
	Lifespan	5–45	19.529 ± 5.032	15.8	[37, 101]
	Survival	53–99.3	78.582 ± 3.821	80	[79, 100, 101]
*Cx. tarsalis*	Development	6.579–15.625	10.117 ± 0.543	9.901	[102]
	Egg viability	43–91.2	78.088 ± 2.06	83	[102–108]
	Lifespan	6–66.7	27.068 ± 1.802	22.55	[104, 107, 108–111]
	Oviposition^a^	36–314.8	150.38 ± 14.748	144	[102–105, 106–109, 112, 113]
	Survival	1–93.2	64.321 ± 3.279	70	[100, 107, 110]

Development = immature development time in days; egg viability = percent of eggs that hatched; lifespan = adult lifespan in days; oviposition = measure of fecundity in number of eggs per egg raft; survival = percent survived to adulthood

^a^Values for other units of oviposition reported (number of egg rafts per female and number of eggs per female per lifespan) are given in Additional file 1: Dataset S1

*Culex pipiens* egg viability was observed to be temperature dependent by two groups [71, 72]. Spanoudis et al. [71] reported that egg hatching remained relatively steady at 70–81% at 15–27.5 °C, then fell off at 30 °C and ceased at 32.5 °C. Oda et al. [72] reported maximum egg viability at 21–25 °C.

Several research groups reported that mean adult female lifespan of laboratory-raised *Cx. pipiens* becomes shorter as ambient air temperature becomes hotter [30, 37, 70–73]. They found that the range of 28–32.5 °C is a maximum temperature where the mosquito lifespan is shorter than WNV extrinsic incubation period or EIP (transit of the virus through the mosquito), thus making transmission impossible at that temperature. WNV external incubation period in *Cx. pipiens* is 16–25 days [74].

Ciota et al. [37] found on the other hand that lifespans of field populations tended to exceed lifespans of laboratory populations. Similarly, [75] observed that under simulated field conditions, adult female *Cx. pipiens* survived for 120 days on average in winter/spring and 80 days on average in summer/autumn, far outliving their constant-temperature counterparts.

Four groups conducting studies spanning 39 years found that *Cx. pipiens* produced the maximum number of egg rafts in the 20–24.6 °C air temperature range [71, 74–77].

Experimental studies are remarkably unanimous in their reports that the maximum survival of *Cx. pipiens* to adulthood happens at 25 °C [71, 79, 82, 83]. *Culex pipiens* development has an inverted U-shaped dependence on temperature. Survival at < 10 or > 33 °C is rare [83].

#### *Culex quinquefasciatus*

Of the 18 studies on *Culex quinquefasciatus* that met our inclusion criteria, most focused on development and survival. The mean development time was 11.403 ± 1.04 days, which was comparable to the other three species examined in this study (Table 2). The fastest observed development rate was 2.703 days at 32 °C [84], and the slowest was 31.25 days at 15 °C [89]. Though most studies reported a linear relationship between temperature and development, this is only true within the survivable temperature range. The minimum threshold for development for this species appears to be between 11 and 18 °C [37, 84, 86, 93, 114]. The maximum threshold is more complex; it appears to be between 34 and 38 °C [84, 86, 93, 114]. Khan and Hossain [87] found that larval *Cx. quinquefasciatus* could survive exposure to 40 °C conditions for 4 h and in fact exhibited shorter development times proportional to the length of exposure. Therefore, it appears that increasing temperature has an inverse effect on development time in *Cx. quinquefasciatus* up until the thermal maximum, the exact value of which is unclear.

According to [93], the optimal temperature range for development in *Cx. quinquefasciatus* is 24–28°C. This is consistent with findings nearly 30 [89] and 50 years later [37, 84]. This is because very low temperatures inhibit development and very high temperatures greatly increase mortality [37, 84, 86, 89, 93, 114].

The lowest temperature at which eggs could hatch was 15 °C [89]. The highest temperature where viable eggs were recorded was 40 °C [87]. Rayah and Groun [114] determined that the ideal temperature for egg viability was 32 °C.

The average adult lifespan for *Cx. quinquefasciatus* across all studies and temperatures was 32.7 days, which was the longest overall of any of the three *Culex* species (Table 2). There was consensus in the literature that lifespan decreases with increasing temperature. The longest recorded adult lifespan was 103.5 days for a mated and blood-fed female at 15 °C [94]. The shortest was 12.2 days for a female at 39 °C [95].

Egg production, or oviposition, was highly variable. Jordan [97] recorded a range of 25–192 eggs per egg raft across different blood-feeding types and temperatures. More recently, 150–200 eggs per egg raft were recorded under standard conditions of 27 °C [90]. Across all included studies, the average oviposition was 131 eggs per egg raft (Table 2). Shriver and Bickley [93] found that egg production increased with temperature up to 35 °C. In direct contradiction to this, however, [94] found 30 °C to be the critical maximum temperature, with reproductive rates highest between 20 and 27 °C and lowest at 30 °C. There are many different units in which oviposition and fecundity are reported, which makes synthesizing the effect of temperature on this parameter difficult.

The mean percent survival to adulthood for *Cx. quinquefasciatus* was 60.65 ± 3.42 across all included studies, which was the lowest overall of the four species (Table 2). Reporting in the literature is somewhat conflicting as to the effect of temperature; some report a positive linear relationship [72], but the consensus appears to be that immature survival decreases at high temperatures [37, 86, 88, 89, 91, 95, 99]. Ciota et al. [37] found immature survival to be highest at the lowest temperature they tested (16 °C), whereas [89] found that it peaked at 25 °C and was less than 50% at both < 15 °C and > 34 °C.

#### *Culex restuans*

We found two experimental studies on *Cx. restuans* development time and survival rate that were published before the introduction of WNV to North America [79, 100]. Both laboratory studies were motivated by encephalitis outbreaks; both found that adult *Cx. restuans* development time decreased with increasing temperature. Although separated by 10 years, both studies found that larval density rather than temperature was most impactful on the proportion of larvae surviving to adulthood.

It is apparent that the lifespan of the adult *Cx. restuans* is shorter than that of the other three species reviewed (mean 19.5 days; median 15.8 days; see Table 2). Experimental life trait data are sparse for this species, and no data were available for oviposition or egg viability.

We found two experimental studies on *Cx. restuans* that were published after the introduction of WNV to North America [37, 101, 115]. Reiskind et al. [115] found that the number of egg rafts per container for *Cx. restuans* varied from 0 to 50 during May/June 2002 and that these oviposition numbers were independent of nutrient concentration. Although they did not record temperatures, their findings are mentioned here due to the lack of oviposition data in the literature for this species.

One study [37] was motivated by the prediction that global warming will increase mean temperatures by 2–4 °C in the next century [116] and by the need to predict how climate influences disease vectors. They observed that adult lifespan decreased with increasing temperature and that, of three species studied, *Culex restuans* was most sensitive to temperature. They also noted that *Culex restuans* was exceptionally difficult to colonize. Muturi et al. [101] were interested in the effects of the pesticide malathion. They found that temperature, not malathion, impacted development and lifespan. Of the three temperatures they studied (20, 25 and 30 °C), at 25 °C development time was minimized, and lifespan was maximized.

#### *Culex tarsalis*

Twelve studies met our inclusion criteria for *Culex tarsalis*, which is the main vector for WNV in the northern Great Plains region [117] and the primary vector for Western Equine Encephalitis Virus (WEEV) in Western North America [109].

In general, the development time of immature mosquitoes had an inverse relationship with temperature whereas the adult mortality rate had a negative nonlinear association with temperature (Fig. 3). These trends were consistent across all four species considered in this review. Thermal stress < 12 °C and > 32 °C is associated with a high rate of mortality for *Cx. tarsalis* larvae [102].

The development rate of *Cx. tarsalis* is influenced by temperature [102]. Milby and Meyer [102] found that the development rate of *Cx. tarsalis* larvae raised in conditions with fluctuating water temperatures (where the mean of the temperatures was equal to the constant laboratory temperature) was not significantly different from the observed development rates with constant temperatures in laboratory [102].

Temperature and crowding were shown to induce significant stress in immature Cx. *tarsalis* [104]. This stress was expressed in the form of reduced survivorship, delayed development, reduced adult wing length, and altered sex ratios [104].

Bock et al. [111] found that life expectancy of adult male *Cx. tarsalis* in laboratory conditions was notably longer (29 days) than that of four other species of mosquitoes, with the next longest life expectancy of 14.8 days for *Culex tritaniorhynchus* Giles.

The values included in this review are for disease-free mosquitoes. The values in this review may not be suitable for infected mosquitoes as some studies demonstrate varying parameter values for infected vs. non-infected mosquitoes. Mahmood et al. [109] found that the lifespan for female *Cx. tarsalis* infected with WEEV was shortened compared to uninfected *Cx. tarsalis.* In contrast, [112] found that WNV infection in *Cx. tarsalis* did not significantly alter the life expectancy of mosquitoes but did cause reduced fecundity in the first gonotrophic cycle.

### Analyses of literature-derived data on *Culex* species’ response to temperature

The extracted data for each parameter-species pair and corresponding best fit model are shown in Fig. 3. Overall, we found that the development rate for all four *Culex* species increased with temperature within the bounds of critical minimum and maximum limits, whereas lifespan decreased with temperature for all species. All species except *Culex restuans* had a unimodal relationship between survival and temperature, where survival peaked between 20 and 28 °C depending on the species (Fig. 3). Similarly, all species showed a unimodal relationship between egg viability and temperature, with the exception of *Cx. restuans*, which had no data. *Culex tarsalis* had the highest oviposition on average, but the range of response was extremely variable; between 26 and 27 °C, the number of eggs per egg raft ranged from 36 to 315. The other species were similar, again excluding *Cx. restuans* because of complete lack of data (Fig. 3).

Overall, *Cx. pipiens* had the steepest regression lines for development and lifespan with respect to temperature, and a relatively broad range for thermal survival tolerance, including two data points ≤ 10 °C. *Culex quinquefasciatus* had the highest overall variability in response, particularly in terms of survival at optimal temperatures. The data for *Cx. pipiens* also had high variability, especially for survival and development at optimal temperatures (Fig. 3).

**Fig. 4:**
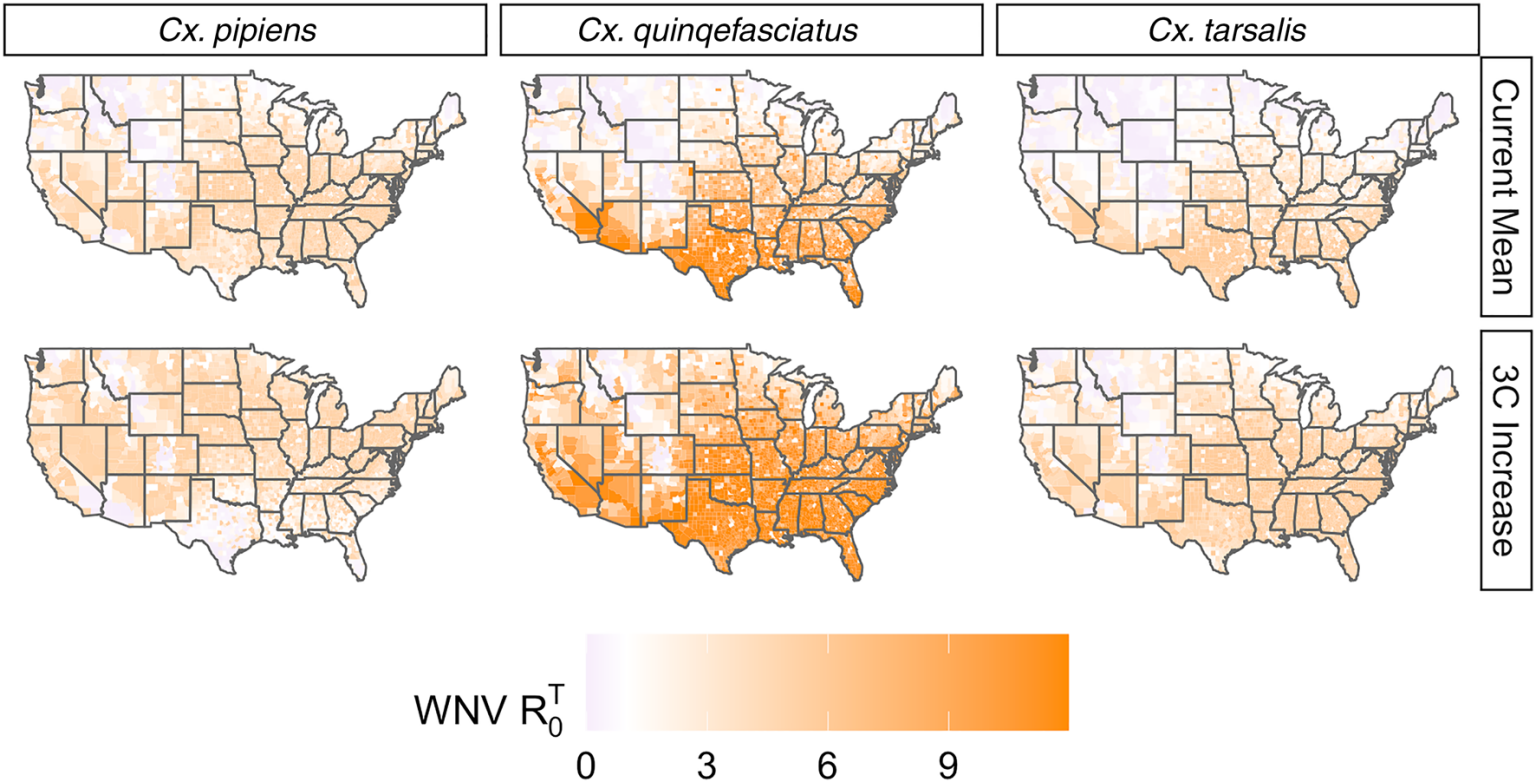
Temperature-dependent WNV mosquito $R_0^T$ for *Culex pipiens* (left column), *Cx. quinquefasciatus* (center column), and *Cx. tarsalis* (right column) under average seasonal temperature conditions from 2010–2020 (top row) and under a 3 °C increase scenario (bottom row). Note that several sources of variability exist in the underlying data which may result in unquantified uncertainty

For West Nile virus mosquito $R_0^T$ potential we created $R_0^T$ temperature-dependent maps informed by empirical data from the literature for *Cx. pipiens, Cx. quinquefasciatus*, and *Cx. tarsalis* (Fig. 4 left to right), excluding *Cx. restuans* because of lack of data. These maps are based on the mean temperature in each state from May to September of 2021 (Fig. 4 top row) and a 3 °C constant increase of those temperatures (Fig. 4 bottom row). From current temperature conditions to the projected 3 °C increase, the following broad changes can be observed: (i) with *Cx. pipiens* as the vector, mosquito WNV transmission potential would increase across the northern third of the US, remain approximately static in much of the Midwest, and decrease in several southern states; (ii) with *Cx. quinquefasciatus* as the vector, mosquito WNV potential would increase in almost all states, especially in the southern US; (iii) with *Cx. tarsalis* as the vector, mosquito WNV potential would increase in the northern two-thirds of the US, while decreasing in Arizona, Texas, Oklahoma, Louisiana, Arkansas, Mississippi, Alabama, Georgia, and Florida.

The impact of state-by-state current temperature on mosquito $R_0^T$ potential varied by *Culex* species. Under 2021 May–September mean temperature conditions, almost every state had a $R_0^T > 1$ for *Cx. pipiens*, while for both *Cx. quinquefasciatus* and *Cx. tarsalis*, most of the Northern states had an $R_0^T < 1$ (Fig. 4). In addition, WNV risk was especially high in the far southern states (i.e. Texas, Florida, Arizona) for *Cx. quinquefasciatus.*

The impact of a 3 °C constant temperature increase on mosquito $R_0^T$ potential also varied substantially by *Culex* species. For *Cx. pipiens* and *Cx. tarsalis*, potential risk decreased such that $R_0^T 1$ for the far southern states, while $R_0^T$ increased to greater than one for most northern states. In contrast, potential risk for *Cx. quinquefasciatus* increased for all states.

## Discussion

In the context of a rapidly changing climate, understanding how environmental factors impact mosquito population dynamics is essential in the fight to mitigate mosquito-borne diseases. Since the introduction of WNV to the US, concentrated research efforts have produced a wealth of data on the effects of temperature on mosquito life history traits in this context. However, to produce accurate disease forecasts that capture biological heterogeneity in input parameters, a detailed understanding of the conclusive patterns as well as the uncertainties within these relationships is needed. To this end, we systematically reviewed literature from 1950 to 2022 on the effects of temperature on five experimentally quantifiable key life history traits in *Culex pipiens, Cx. quinquefasciatus, Cx. tarsalis*, and *Cx. restuans*, four important vector species for WNV in the US. We identified 45 research articles that met our criteria for inclusion. Using data from these papers and data compiled in [11], we constructed mosquito WNV $R_0^T$ potential transmission risk maps with annual mean monthly temperature data from May–September, 2010–2020, and under a 3 °C constant projected increase as a use case scenario and illustration of the utility of the data collected in this study.

We found consensus in the literature that immature development rate is positively correlated with temperature in all four species, while adult lifespan is negatively correlated with temperature in all four species. We applied linear fits to both these traits, consistent with previous studies [67] though in reality the relationships are slightly more complex because of critical minimum and maximum thermal thresholds [37]. Survivability differed among species; for example, *Cx. restuans* can stay alive within a narrower temperature range than the other species, while *Cx. quinquefasciatus* can tolerate higher temperatures than the others. We applied quadratic fits to egg viability and survival, which both exhibited an inverted U-shaped relationship to temperature, consistent with optimal ranges previously reported [11, 37].

The cumulative effects of the five life history traits on mosquito WNV transmission potential were nonlinear, and some contradictory. We speculate that with increased temperatures, faster development times could increase WNV transmission, while shorter lifespans could decrease transmission windows. Shocket et al. [11] established an optimal WNV transmission temperature range of 24–25 °C, which falls on the lower end of optimal ranges for many life history traits across species. However, Vogels et al. [118] predicted widespread WNV establishment at a minimum annual average of 28 °C, which falls at the peak of many life traits. More recently, Di Pol et al. [119] found that WNV may become established in *Culex* vectors between 14 and 34.3 °C, with an optimal temperature suitability of 23.7 °C. We suggest that some of the variability in these results could be improved by incorporating different *Culex* species types at infection into transmission risk models. An additional factor supporting the idea that hotter temperatures could support increased WNV transmission is that the EIP for other pathogens has been shown to decrease with temperature, resulting in more efficient virus transmission [58]. Even with lower egg viability and survival rates, decreased EIP combined with mosquitoes’ many mechanisms for resiliency and adaptation could create unexpectedly high WNV incidence at high temperatures.

Our mosquito $R_0^T$ potential transmission maps incorporate the distributions of all five life traits from the literature and illustrate consequential differences in the overall way different *Culex* species’ responses to temperature can impact the spread of WNV. Under a 3 °C increase, our projected northward shift of *Cx. pipiens*-informed risk is consistent with evidence of northward range expansion and low tolerance for hot and dry conditions [20]. The nearly opposite shift of *Cx. quinquefasciatus*-informed risk is consistent with their preference for hot climates and urban areas [23, 120, 121], as well as their potential for extreme heat tolerance [86]. *Culex tarsalis*-informed risk shrank in southern/southeastern states and grew in central, western, and northern states, which matches projected habitat suitability for the species and its preference for rural agricultural land [23, 50, 117, 122].

We limited our review to the relationships between temperature and five life history traits of four *Culex* species; however, there are other important factors not considered here that can have strong impacts on *Culex* populations and WNV transmission. Some abiotic factors are relative humidity, rainfall, daily temperature fluctuation, and photoperiod. Some of the biological factors important to this topic are density dependence, intra- and inter-species competition, vegetation and land cover, body size, biting rates, and age-specific mortality rates [123].

Our review focused on four *Culex* species as WNV vectors, although other mosquito vectors and transmission routes exist. Examples of other vectors not considered in this study are *Culex nigripalpus* [23] and *Aedes albopictus* [124]. Additionally, though vertical transmission of WNV exists and is tied to climatic variation [59, 60], we limited our consideration to horizontal transmission.

We found that experimental studies of temperature-dependent traits in *Cx. restuans* were sparse relative to the other three species reviewed (Fig. 3, Table 2). A comprehensive analysis of the lack of *Cx. restuans* studies is outside the scope of this article. However, we speculate that *Cx. restuans* has been less studied because (i) its role in WNV transmission has been more recently identified relative to the others and (ii) *Cx. restuans* has a history of being difficult to distinguish morphologically from *Cx. pipiens* [125, 126].

There are several sources of variability in the data summarized here. The studies reviewed took place in different geographic regions with different climates and ecologies, during different years or even decades. Mosquito strains originated from a broad range of locations. Some experiments were conducted in the field, while others were conducted under laboratory conditions and others did not clearly indicate their settings. Studies were carried out using a variety of methodologies. Furthermore, temperature dependence of life traits was measured regarding air temperature or water temperature, or not specified. These underlying sources of variation may result in the presence of unquantified uncertainty in our distribution maps (Fig. 4).

Studies that evaluate the impact of temperature on mosquito life traits often equate or do not differentiate between water and air temperature, though water temperature is most used for parameters involving immature stages, and air temperature is most used for parameters involving adult stages. In our literature review we reported both air and water temperatures, depending on the methods used in the studies (Fig. 3, see Additional file 4: Dataset S4). In our species-specific mosquito $R_0^T$ potential estimates, we did not distinguish between air and water temperatures, according to precedent [49]. These are distinguished in Additional file 4: Dataset S4. Arjunan et al. [84]. Furthermore, since the majority of the papers that fit our inclusion criteria were laboratory studies, the data here cannot be taken to wholly represent field mosquito populations that transmit WNV under actual conditions [37]. We distinguished laboratory strains from field-caught mosquito populations (Fig. 3, see Additional file 4: Dataset S4).

WNV mosquito vectors possess a robust pool of genetic diversity because of overlapping ranges and hybridization [69, 127]. An important concept to consider when evaluating effects of climate on mosquito populations is that many species, especially those prone to rapid adaptation and hybridization such as *Culex quinquefasciatus*, probably engage in tradeoffs between life history traits under temperature stress. This is certainly connected to evolutionary seasonal survival strategies like diapause and quiescence [128]. *Culex pipiens, Cx. restuans*, and *Cx. tarsalis* all engage in adult female diapause [129, 130], whereas *Cx. quinquefasciatus* lacks the capacity for dormancy [131]. However, *Cx. pipiens-Cx. quinquefasciatus* hybrids are capable of diapause [132]. Further studies are needed to evaluate tradeoffs between life history traits in different species such as development, survival, and reproductive output at different temperature ranges, coupled with hybridization, adaptation to stress, disease transmission, and ultimately the heterogeneity present in all these factors and their interactions.

Accurate parameterization of complex biological traits is not only relevant to WNV, but also to other vector-borne disease systems. For example, dengue is often modeled with *Aedes aegypti* as the sole vector although others are known, and similarly, Lyme disease is often modeled with *Ixodes scapularis* as sole vector, although at least four species are thought to be central to its transmission.

Given the multidimensional variability and potential interactions of the data found in this review that were not accounted for, possible next steps to refine the model presented here could include using random intercepts and/or interaction terms.

Another proposed avenue for understanding and incorporating uncertainty related to the effects of temperature on biological parameters broadly influencing disease risk is to use a Bayesian approach to identify certain parameters and temperature ranges that dominate uncertainty in thermal response. This has been done for malaria [133]. Most notable in that study were biting rate from 15 to 25 °C, fecundity across all temperatures, and mortality/survival from 20 to 30 °C. Applying this method to the WNV system and more broadly analogous vector-borne disease systems could be a practical way to approach bridging the gaps between mechanistic models, field transmission, and the life history trait and temperature variation investigated in the present scoping review.

## Conclusion

In this review, we assembled and assessed experimental data from literature published between 1950 and on the effects of temperature on key life history traits in *Culex* mosquito vector vector species important to the WNV transmission cycle. In the context of climate change, it is essential to understand and apply these relationships accurately in dynamic predictions of diseases such as WNV. Future models will ideally be able to incorporate more precise representation of these species’ temperature-dependent dynamics in predicting population and disease transmission dynamics. The contents of this review provide a valuable resource toward that goal.

## Supplementary Information

**Additional file 1: Dataset S1.** Combined and cleaned raw data obtained from the literature used to construct Table 2, Figs. 3, and 4; includes oviposition data in units other than eggs per egg raft.

**Additional file 2: Dataset S2.** Full details of the review broken down by parameter and species, including data and notes.

**Additional file 3: Dataset S3** Literature search details including full list of search terms and breakdown of number of initial hits and records included after each filtering step.

**Additional file 4: Dataset S4.** Information on mosquito strain origin and temperature measurement types.

**Additional file 5: Table S5.** Descriptions of temperature-dependent functions fitted to the raw data and AICc values.

## Data Availability

All data generated or analyzed during this study are included in this published article and its supplementary information files.

## Abbreviations

*Cx.*: Culex
WNV: West Nile virus
$R_0^T$: Temperature-dependent mosquito type reproduction number
EIP: Extrinsic incubation period
AICc: Second-order Akaike information criterion
