# Supplementary material for: Scoping review of Culex mosquito life history trait heterogeneity in response to temperature
Source: Parasit Vectors. 2023 Jun 14;16:200. doi: 10.1186/s13071-023-05792-3 (PMC10265793; doi:10.1186/s13071-023-05792-3)
Supplement: Supplementary file 5 — Additional file 5: Table S5. Descriptions of temperature-dependent functions fitted to the raw data and AICc values. [file 13071_2023_5792_MOESM5_ESM.docx]

**Supplementary File 5:** Fitted temperature-dependent parameter functions and AICc values for literature-derived data that had not already been fit in Shocket et al (2020)

Fitted coefficient values for the linear model: $\beta_{0} +\beta_{1}T$where $T$ is temperature in C

| Trait | Species | $\beta_{0}$ | $\beta_{1}$ |
| --- | --- | --- | --- |
| Development (days) | pipiens | 48.37773 | -1.1890889 |
|  | quinquefasciatus | 28.11306 | -0.6103044 |
|  | restuans | 41.48844 | -1.2639824 |
|  | tarsalis | 21.26403 | -0.4657052 |
| Lifespan (days) | pipiens | 124.91681 | -3.8549414 |
|  | quinquefasciatus | 102.06115 | -2.5730726 |
|  | restuans | 68.86312 | -1.9292842 |
|  | tarsalis | 70.77825 | -1.8171857 |

Fitted coefficient values for the quadratic model: $a + bT + cT^{2}$ where $T$ is temperature in C

| Trait | Species | $a$ | $b$ | $c$ |
| --- | --- | --- | --- | --- |
| Egg viability (%) | pipiens | -71.22262 | 16.568571 | -0.44495238 |
|  | quinquefasciatus | -81.61029 | 11.339578 | -0.1956908 |
|  | tarsalis | -396.52791 | 38.332510 | -0.76754386 |
| Survival (%) | pipiens | -72.84127 | 13.186251 | -0.29073926 |
|  | quinquefasciatus | -85.14358 | 12.415546 | -0.24547267 |
|  | restuans | 130.13329 | -1.855478 | -0.02087542 |
|  | tarsalis | -43.46771 | 8.520785 | -0.15529665 |

| Trait | Species | Quadratic | Briére | Linear |
| --- | --- | --- | --- | --- |
| Development (days) | pipiens | **-169.867** | -168.473 | -169.588 |
|  | quinquefasciatus | -90.2519 | -87.842 | **-92.336** |
|  | restuans | -80.596 | -75.937 | **-82.897** |
|  | tarsalis | -91.137 | -87.243 | **-91.826** |
| Lifespan (days) | pipiens | **249.657** | 298.225 | 258.192 |
|  | quinquefasciatus | 251.238 | 277.090 | **249.463** |
|  | restuans | 78.693 | 122.460 | **64.700** |
|  | tarsalis | 555.628 | 646.757 | **554.772** |
| Egg viability (%) | pipiens | **64.480** | 89.393 | 76.034 |
|  | quinquefasciatus | 92.276 | 106.219 | **87.860** |
|  | tarsalis | **243.569** | 245.755 | 259.366 |
| Survival (%) | pipiens | **348.559** | 355.733 | 372.774 |
|  | quinquefasciatus | **517.275** | 525.422 | 525.739 |
|  | restuans | 137.308 | 157.499 | **133.858** |
|  | tarsalis | **373.357** | 378.594 | 375.003 |

S5: Table 1. AICc values for each model, the lowest (and therefore best) AICc values for each species and life history trait are bolded. We used the model that was best across all species for each trait as previous literature has fit the same model to all species for a given trait.
